# Evaluation of Electrocardiographic Changes after Arterial Switch Operation

**Published:** 2014-09-01

**Authors:** Hamid Amoozgar, Ahmad Ali Amirghofran, Shirvan Salaminia, Sirous Cheriki, Mohammad Borzoee, Gholamhossein Ajami, Farah Peiravian

**Affiliations:** 1 Cardiovascular Research Center, Shiraz University of Medical Sciences, Shiraz, IR Iran; 2 Department of Cardiac Surgery, Shiraz University of Medical Sciences, Shiraz, IR Iran; 3 Division of Pediatric Cardiology, Department of Pediatrics, Shiraz University of Medical Sciences, Shiraz, IR Iran; 4 Department of Pediatric, Islamic Azad University, Kazerun Branch, Kazerun, IR Iran

**Keywords:** Transposition of Great Vessels, Switch, Surgical Procedures, Follow-up Studies, Electrocardiography

## Abstract

**Background:**

Transposition of Great Arteries (TGA) is a serious congenital heart disease which can be accompanied by good outcomes with anatomic correction within the first few weeks of life.

**Objectives:**

The present study aimed to evaluate electrocardiographic changes in the children with uncomplicated Arterial Switch Operation (ASO).

**Patients and Methods:**

Twelve lead electrocardiograms were obtained from thirty-three patients with TGA after ASO. Then, the patients’ QT-dispersion and P-wave dispersion were compared to those of 33 age- and gender-matched individuals as the normal control group. Both groups were also evaluated by M-mode echocardiography. Student’s t-test and Pearson correlation were used to analyze the data. Besides, P < 0.05 was considered as statistically significant.

**Results:**

The mean age of the patients and the control group was 41 ± 3.7 and 40.12 ± 4.2 months, respectively. Comparison of P wave, T wave, QRS complex, PR interval, QT segment, and corrected QT segment showed significant differences in the frequency of inverted T wave in pericardial leads [V3, V4, V5, and V6] (P = 0.004; more in patients), P wave amplitude in lead II (P < 0.001; more in patients), R wave amplitude in V1 (P = 0.016; smaller in patients), R and S waves amplitude in V6 (P = 0.004 and P = 0.001; taller in patients), corrected QT segment (in lead V5; P < 0.0001: prolonger in patients), and PR interval (P = 0.001; prolonger in patients). However, no significant differences were found regarding the vector axis and heart rate. Right bundle branch block (18% vs. 0%), Bifascicular (3% vs. 0%), and first-degree blocks (15% vs. 0%) were significantly more in the patients. Besides, the PR interval was longer in the corrected complex TGA (146 ± 24.4 vs. 127.7 ± 23.1, P = 0.001). Moreover, R/S ratio in lead V1 was significantly prolonger, among the patients (2.86 ± 3.35 vs. 0.706 ± 0.53, P = 0.002). Nonetheless, no significant was observed between the patients and controls concerning the mean of QT dispersion. On the other hand, the two groups were significantly difference in terms of P wave dispersion (25.7 ± 13.8 vs. 33.74 ± 12.9, P = 0.024).

**Conclusions:**

In this study, first-degree block and right bundle branch were detected in the operated patients with TGA. Increased P dispersion in these patients may increase the risk of atrial arrhythmia. Thus, long-term follow-up is necessary in these patients.

## 1. Background

Through the past decades, anatomical correction has become the treatment of choice for simple Transposition of the Great Arteries (TGA) as well as for transposition with ventricular septal defect. The prominent concerns about this operation are the ideal age for surgery, late ventricular function, neo-aortic valve competence, late coronary ostial stenosis, growth of arterial anastomoses, and frequency of postoperative arrhythmias (1). Cardiac arrhythmias are the common postoperative sequelae of the intra-arterial repair of d-transposition of the great arteries (2).

Multiple studies have reported the electrophysiological abnormalities associated with these types of surgical repair. Significant cardiac arrhythmias, including sick sinus syndrome and supraventricular arrhythmias, especially atrial flutter, have been reported (3) in as many as 50% of these patients after the Mustard repair. Senning repair has also been increasingly utilized, but studies have shown arrhythmias similar to those detected after the Mustard repair. Hence, Arterial Switch Operation (ASO), first reported by Jatene in 1975, has become the procedure of choice for the patients with TGA and Taussig-Bing anomaly (4). ASO has a low incidence of arrhythmias, at least in short-term. However, there are few reports that have focused on post-operative arrhythmias during mid- and long-term follow-up. As follow-up lengthens, any advantage of anatomic correction with respect to arrhythmias should be confirmed. Many of the recent studies are about the short-term arrhythmic sequelae of such patients who have stayed alive for more than one year.

## 2. Objectives

Therefore, the present study aims to evaluate electrocardiographic changes after midterm follow-up in transposition of great vessels following ASO.

## 3. Patients and Methods

### 3.1. Patient Population

This case control study was conducted on thirty three children who were operated by a single surgeon in two hospitals (Shahid Faghihi and Dena hospitals, Shiraz, Iran) between January 2005 and December 2011. Written informed consents were obtained from all the parents, and the study was approved by the Ethics Committee of Shiraz University of Medical Sciences, Shiraz, Iran.

### 3.2. Control Group

The control group included the children with the same age and sex as the patients who had no cardiac diseases according to history, echocardiography, and electrocardiography and had referred to the clinic for screening. All the children with any history or abnormal echocardiographic findings were excluded from the study.

### 3.3. Electrocardiography

A twelve leads electrocardiographic study was done for both groups (by 12 lead digital electrocardiogram, Esaote P8000). The electrocardiographic parameters of P-wave duration, axis, and amplitude [in lead II], PR interval [in lead II], QT and corrected QT segment (QTC according to Bazett's formula), QRS complex duration [in V5], R-wave amplitude in leads V1 and V6, and S-wave amplitude in leads V1 and V6, and also if inverted T-wave was seen in three or more pericardial leads, were recorded. In addition to these ECG measurements, P wave dispersion and QT segment dispersion were also measured for both groups. In order to measure QT and P-wave dispersion, at first both parameters were measured in 12 leads and then the difference between the longest and shortest amounts were assigned as P-wave dispersion and QT dispersion, respectively. Afterwards, the two study groups were compared regarding the measured parameters.

### 3.4. Echocardiography

A 2-dimensional echocardiography, including M-mode, was done for both groups. The preoperative data included age, sex, age at operation, present age, body weight, hemoglobin content, and any previous cardiac operation or interventional treatment. All these data were recorded in a special form that was structured for each patient. No preoperational angiography was done, except for one patient with preoperational atrial septostomy.

### 3.5. Arterial Switch Operation Description in Short

ASO was performed under systemic anesthesia, using low-flow (i.e., 50 - 100 mL/kg/ min) hypothermic (range, 28° to 32°C) cardiopulmonary bypass. After cannulation and before starting cardiopulmonary bypass, ductus arteriosus was ligated and divided and then division of aorta and pulmonary artery was done. After transfer of coronary arteries, reconstruction of pulmonary artery was done with autologous pericardium in all the patients. In the patients with ventricular septal defect, Gortex patch was used to repair the ventricular septal defect.

### 3.6. Palliative Operations

In this study, three patients (1 patient with TGA, 2 patients with TGA plus ventricular septal defect) underwent pulmonary artery banding operation plus systemic to pulmonary shunt operation. Later on when these patients were suitable for operation, they underwent ASO. Except for these three patients, all the other patients had a single stage total correction.

### 3.7. Statistical Analysis

The statistical analyses were performed using the SPSS statistical software, version 16 (SPSS, Inc. Chicago, IL). The data were expressed as mean ± one standard deviation. Student’s t test was used to compare the mean values of the patients and the control group with the probability values beingstatistically significant at < 0.05 levels. In addition, Pearson correlation and linear regression were used to evaluate the correlation between the variables.

## 4. Results

The mean age of the patients and the control group was 41 ± 3.7 and 40.12 ± 4.2 months, respectively. In both groups, 20 subjects were male and 13 ones were female. The patients’ mean age at the time of operation was 23 days.

### 4.1. Electrocardiographic Findings

Comparison of P wave, T wave, QRS complex, PR interval, QT segment, and corrected QT segment between the patients and the control group showed significant differences in the frequency of inverted T wave in pericardial leads [V3, V4, V5 and V6] (P = 0.004; more in the patients), P wave amplitude in lead II (P < 0.001); more in the patients), R wave amplitude in V1 (P = 0.016; smaller in the patients), R and S waves amplitude in V6 (P = 0.004 and 0.001; taller in the patients), corrected QT segment (P < 0.001; prolonger in the patients), and PR interval (P = 0.001; prolonger in the patients).

However, no significant difference was found regarding vector axis and heart rate. The patients’ right bundle branch block, bifascicular, and first-degree blocks were significantly higher compared to the control group (P < 0.05). Yet, no left bundle branch block and 2nd and 3rd degree blocks were seen. Considering the complexity of TGA, only the PR interval was longer in complex TGA (P = 0.049). Moreover, no statistically significant correlations were observed between the electrocardiographic parameters and the preoperational parameters of age at operation, operation time, pump time, aortic cross clamp time, hospital stay, and need for intensive care unit. Nevertheless, R/S voltage ratio in lead V1 was significantly more among the patients (2.86 ± 3.35 vs. 0.706 ± 0.53; P = 0.002), but no difference was observed concerning lead V6.

The study results revealed some differences between the measured parameters in the patients and the general population normal values. R wave in lead V1 was taller than normal. Besides, both Pamplitude and PR interval were more than normal values. However, no significant difference was observed between the two groups regarding the other parameters. Nonetheless, due to lack of definite normal values, no comparisons were made with normal values for some variables, including P wave dispersion, QT dispersion, and R/S slope.

The results also indicated no significant difference between the patients and the control group regarding the mean of QT dispersion. On the other hand, the two groups were significantly different with respect to P wave dispersion (25.7 ± 13.8 vs. 33.74 ± 12.9; P = 0.024). These ECG findings have been presented in Table 1.

**Table 1.** Comparison of the Measured Electrocardiographic Parameters between the Patients and Controls

Item	Patients (Mean ± SD)	Normal Group (Mean ± SD)	P
**QT dispersion (ms)**	43.9 ± 2.12	46.95 ± 2.20	0.67
**P dispersion (ms)**	25.7 ± 13.80	33.74 ± 12.9	0.02
**Vector axis according to normal value for age**			
**1) Normal**	24 (72%)	33	
**2) Left**	1 (3%)	0	
**3) Right**	7 (21%)	0	
**4) Supreme right**	1 (1%)	0	
**P wave duration (ms)**	95.28 ± 13.98	95.75 ± 17.80	0.56
**P wave axis (degree)**	43 ± 12.85	41.9 ± 15.82	0.77
**P wave amplitude in lead II (mv)**	0.19 ± 0.05	0.145 ± 0.04	< 0.001
**R in V**_**1**_ ** (mv)**	0.66 ± 0.54	0.40 ± 0.20	0.016
**S in V**_**1**_ ** (mv)**	0.48 ± 0.51	0.72 ± 0.40	0.050
**R in V**_**6**_ ** (mv)**	1.49 ± 0.55	1.08± 0.41	0.004
**S in V**_**6**_ ** (mv)**	0.37± 0.33	0.15 ± 0.10	0.001
**PR interval (ms)**	137.5 ± 25.18	106.23 ± 21.30	< 0.001
**QT (ms)**	310.57 ± 32.84	307.67 ± 64.37	0.83
**Corrected QT [QTC] (ms)**	440.29 ± 19.83	423.62±15.58	0.001
**QRS amplitude lead V5 (mv)**	95.00 ± 16.70	87.79±5.91	0.03
**QRS axis (degree)**	67.71 ± 65.75	67.10 ±26.84	0.80
**T wave axis (degree)**	34.178 ± 14.90	32.79±13.00	0.71
**Heart rate (bpm)**	119.96 ± 30.86	107.90±21.82	0.08
**Premature ventricular contraction (PVC)**	0	0	-
**Atrial fibrillation (AF)**	0	0	-
**1** ^**st**^ ** degree block**	5	0	< 0.001
**2** ^**nd**^ ** degree block**	0	0	-
**3** ^**rd**^ ** degree block**	0	0	-
**Right bundle branch block (RBBB)**	6	0	< 0.001
**Left bundle branch block (LBBB)**	0	0	-
**Bifascicular block (BFB)**	1	0	0.051
**Trifascicular block (TFB)**	0	0	-

### 4.2. Echocardiographic Findings

In echocardiographic evaluation, 6% of the patients had mild pulmonary stenosis, while 3% had mild pulmonary insufficiency. Additionally, aortic stenosis and aortic insufficiency of trivial tomild degree were detected in 12% and 12% of the patients, respectively. No significant correlation was found between the echocardiographic and electrocardiographic parameters.

## 5. Discussion

In this study, in addition to routine 12-leads electrocardiographic evaluation, we also compared depolarization and repolarization changes. These measurements included both QT dispersion and P-wave dispersion. According to the results, three study patients had Junctional Ectopic Tachycardia (JET) after anatomic correction (hospital course). Besides, Right Bundle Branch Block (RBBB) and first-degree atrioventricular block were detected in 33% of the patients. The results demonstrated a significant difference between the patients and the control group regarding R and S waves in leads V1 and V6. In addition, QTC and PR interval were significantly longer among the patients compared to the control group. However, no significant difference was found between the two groups concerning QRS axis, P wave axis, P wave duration, heart rate, T wave axis, and QT dispersion. Nevertheless, P wave dispersion and P wave voltage were significantly higher among the patients compared to the control group. Most of our patients (82% vs. 10% in the control group) had inverted T wave in three or more of the precordial leads.

Up to now, a limited number of studies has been conducted on the prevalence and risk factors of arrhythmia after ASO. In a study in Japan, 9.6% of 1-year survivors of ASO had significant arrhythmias. Besides, bradycardia occurred in 22 patients, including complete atrioventricular block in 0.2%, sick sinus syndrome in 0.1, and second- degree atrioventricular block in 0.6%. Additionally, syncope developed in 0.3% with complete heart block and 0.3% with sick sinus syndrome. Supraveutricular tachycardia was seen in 4% of the patients, including paroxysmal supraventricular tachycardia in 2%, atrial flutter in 1%, and atrial fibrillation in 1%. Ventricular arrhythmias also occurred in 2% of the patients, including non sustained ventricular tachycardia in 0.8%, paroxysmal ventricular contractions with couplets in 0.8%, ventricular flutter in 0.3%, and sustained ventricular tachycardia in 0.1%. Death was directly related to arrhythmia in 0.1% of the patient. Moreover, presence of a ventricular septal defect was a risk factor for postoperative arrhythmia (5).

In the literature, electrocardiographic parameters have been evaluated for prediction of risk of arrhythmias and diagnosis of arrhythmias for different cardiac and noncardiac diseases. In an article by Jared et al., P wave dispersion was evaluated as a predictor of atrial fibrillation after different cardiac pathologies and surgeries. They concluded that these indexes were associated with the clinical risk factors for atrial fibrillation (6). In another study, P wave dispersion provided valuable markers for early recognition of high-risk patients for atrial fibrillation, which may guide upstream therapy (7). In the present study, P wave indexes (P wave amplitude, duration, PR interval, and P wave dispersion), except for p wave duration, were significantly higher among the patients in comparison to the normal control group. Considering this difference, we may conclude that our patients with ASO were at a higher risk for arrhythmia compared to the control group.

Overall, this study supported the need for close follow-up of these patients. P wave dispersion is an ECG index. This index has a diurnal variation in healthy subjects, such as being shorter in summer and longer in winter (8). Up to now, the most extensive clinical evaluation of P wave dispersion has been performed for assessment of the risk of atrial fibrillation. Several studies have shown that P wave dispersion has a predictive value for atrial fibrillation in the patients without apparent heart disease, hypertensives patients, those with coronary artery disease, and the patients undergoing coronary artery bypass surgery. Moreover, P wave dispersion has been proven to be a sensitive and specific ECG predictor of atrial fibrillation in various clinical settings (9). Investigations of R wave, S wave, and R/S progression and ratio in leads V1 and V6 have revealed divergent results. In the current study, all these measurements were significantly higher among the patients compared to the normal control group. However, in comparison to general population standards appropriated for age and sex, all these parameters were within the standard ranges.

Therefore, no definite conclusions can be drawn. Furthermore, these findings may be due to either biventricular hypertrophy or disarrangement in electrical conduction. The researchers could find no studies evaluating these parameters as predictors of any specific diagnosis, except for diagnosis of ventricular hypertrophy and sometimes disarrangements in bundle branch conductions.

The results of the present study indicated no significant difference between the patients and the control group regarding QT segment and QT dispersion. Also, all the measurements in both groups were within the standard ranges of general population appropriated for age and sex. However, corrected QT segment was significantly more among the patients compared to the control group, but not different from the normal values. Increased values of QT and QT dispersion are known to be a marker of myocardial instability and result in myocardial vulnerability to serious ventricular arrhythmias (10, 11). Increased dispersion of refractoriness is a consequence of in homogeneity in ventricular repolarization, which is implicated possibly in the generation of reentry circuits, resulting in arrhythmias. Increased QT dispersion has also been correlated with an increased incidence of ventricular arrhythmias in a variety of diseases, such as long QT syndromes, hypertrophic cardiomyopathy, essential hypertension, congestive heart failure, and mitral valve prolapse (12-16). Yet, the researchers could not find any studies specifically investigating these refractoriness parameters in children with congenital heart diseases, especially for transposition of great vessels. In this study, considering the measurements and comparisons with the control group and the general population normal values, these parameters were against the increased risk of ventricular arrhythmias in our ASO patients.

ASO is not free from arrhythmic complications. Longer aortic cross-clamp time and early age of operation have, in general, been found to be the risk factors of rhythm disturbances among pediatric cardiac procedures (17). It is also sensitive to time of the day, season of the year, and even body position (18). Closure of ventricular septal defect has also been implicated as a risk factor (5, 19). Nonetheless, supraventricular tachycardias after ASO are uncommon compared to the Mustard and Senning procedures. However, in our study, none of the measured parameters were correlated to operation age, current age, operation time, pump time, cross clamp time, and complexity of TGA, except for PR interval that had a positive correlation with increase in complex TGA and increase of current age.

It seems that correction of TGA with arterial switch is associated with a low incidence of clinically significant arrhythmias (19). ASO is performed at the level of the great arteries and requires less surgical manipulation of the atria than do the Mustard and Senning inflow correction procedures.

This results in less fibrosis and suture lines that can disrupt the conduction pathways within the heart, and sinus node function to be well preserved. Closure of congenital and iatrogenic atrial septal defects has not been found to be a risk factor for developing rhythm disturbances following ASO (2). Studies reviewing patient series have supported the electrophysiological benefits of the arterial switch over the inflow correction procedures (2). Williams and colleagues found freedom from permanent pacing 15 years post operation to be 98% for ASO and 89% for Mustard and Senning procedures. Although much of studies presented in the literature are about postoperative dysrhythmia in TGA, discussion of dysrhythmia after ASO is very scant, and almost all these studies are about dysrhythmia after either Senning or Mustard operation. This lack of considerable studies on dysrhythmia after ASO may be related to the idea or the fact that post operational dysrhythmia in this group of patients is low or at least not as high as atrial switch. Another reason may be that older children are normally operated through atrial switch rather than ASO.

However, the risk of dysrhythmia after ASO is definitely higher among such patients compared to the general population. This might be due to the fact that these patients have accompanied anatomic defects, such as atrial septal defect and ventricular septal defect. The major cardiovascular surgery, cannulation, and the following scar formation are arrhythmogenic. The nature of pathology and the embryonic defect are also arrhythmogenic. In addition, some of these patients have cardiac dysfunction preoperatively, which could be a source of arrhythmia after the operation.

Retrospective studies have demonstrated a much lower incidence of significant tachy-bradyarrhythmias (19) and atrioventricular conduction abnormalities (20) after anatomic correction. Interpretation of these findings is complicated due to the heterogeneous nature of both the patients and the used surgical techniques. Some of these arrhythmias first occur before the operation (20).

According to our search, no studies have been conducted on QT dispersion and P wave dispersion after TGA and its correction, except for the one performed by Zhi-hong Sun et al. that reported a significant relationship between QT dispersion and sudden cardiac death after ASO (21). In that study, all the patients preserved normal sinus rhythm, but some conduction abnormalities were detected, as well (22).

The results of this study showed some differences between the patients and the control group regarding the electrocardiographic parameters. Moreover, first-degree heart blockand right bundle branch were more common among the patients. Furthermore, corrected QT and Pwave dispersion increased in the patient after ASO and this might increase the chance of arrhythmia. The presence of a ventricular septal defect was also a risk factor for postoperative arrhythmia.
